# Peripheralized sepiapterin reductase inhibition as a safe analgesic therapy

**DOI:** 10.3389/fphar.2023.1173599

**Published:** 2023-05-12

**Authors:** Shane J. F. Cronin, Nick A. Andrews, Alban Latremoliere

**Affiliations:** ^1^ Institute of Molecular Biotechnology Austria (IMBA), Vienna, Austria; ^2^ The Salk Institute for Biological Studies, La Jolla, CA, United States; ^3^ Departments of Neurosurgery and Neuroscience, Johns Hopkins School of Medicine, Neurosurgery Pain Research Institute, Baltimore, MD, United States

**Keywords:** Sepiapterin Reductase, salvage pathways, nociceptors, immune cells, PROTAC, hyaluronic acid, aldoketoreductase, carbonyl reductase

## Abstract

The development of novel analgesics for chronic pain in the last 2 decades has proven virtually intractable, typically failing due to lack of efficacy and dose-limiting side effects. Identified through unbiased gene expression profiling experiments in rats and confirmed by human genome-wide association studies, the role of excessive tetrahydrobiopterin (BH4) in chronic pain has been validated by numerous clinical and preclinical studies. BH4 is an essential cofactor for aromatic amino acid hydroxylases, nitric oxide synthases, and alkylglycerol monooxygenase so a lack of BH4 leads to a range of symptoms in the periphery and central nervous system (CNS). An ideal therapeutic goal therefore would be to block excessive BH4 production, while preventing potential BH4 rundown. In this review, we make the case that sepiapterin reductase (SPR) inhibition restricted to the periphery (i.e., excluded from the spinal cord and brain), is an efficacious and safe target to alleviate chronic pain. First, we describe how different cell types that engage in BH4 overproduction and contribute to pain hypersensitivity, are themselves restricted to peripheral tissues and show their blockade is sufficient to alleviate pain. We discuss the likely safety profile of peripherally restricted SPR inhibition based on human genetic data, the biochemical alternate routes of BH4 production in various tissues and species, and the potential pitfalls to predictive translation when using rodents. Finally, we propose and discuss possible formulation and molecular strategies to achieve peripherally restricted, potent SPR inhibition to treat not only chronic pain but other conditions where excessive BH4 has been demonstrated to be pathological.

## 1 Introduction

The development of new analgesics for chronic pain has suffered many failures, and the main challenges encountered are a relative lack of efficacy, and dose-limiting side effects (Edwards et al., 2016; Edwards et al., 2023). This is particularly true for pharmacological treatments for neuropathic pain, a chronic pain condition that can arise after an injury to the somatosensory system. The first line of medication for neuropathic pain broadly consists of pregabalin/gabapentin, morphine, tramadol, tricyclic antidepressants and carbamazepine. In addition to their modest analgesic efficacy, these drugs are associated with severe central nervous system (CNS) side effects (somnolence, cognitive disruption (described by patients as ‘head in the fog’), memory impairments, lack of general energy), to a point where they cause significant dropout rates. Drugs with opioid activity such as morphine and tramadol also have dependence liability and a range of other serious side-effects. A major difficulty to dissociate efficacy from CNS side effects is that most molecular targets for reducing neuropathic pain are located in the brain (mu opioid receptor (MOR), α2δ1 channels, noradrenaline- and serotonin-re-uptake inhibitors heavily expressed in brain and spinal cord). There is therefore a need to identify new analgesic drug targets with better efficacy/side effects profiles.

**FIGURE 1:**
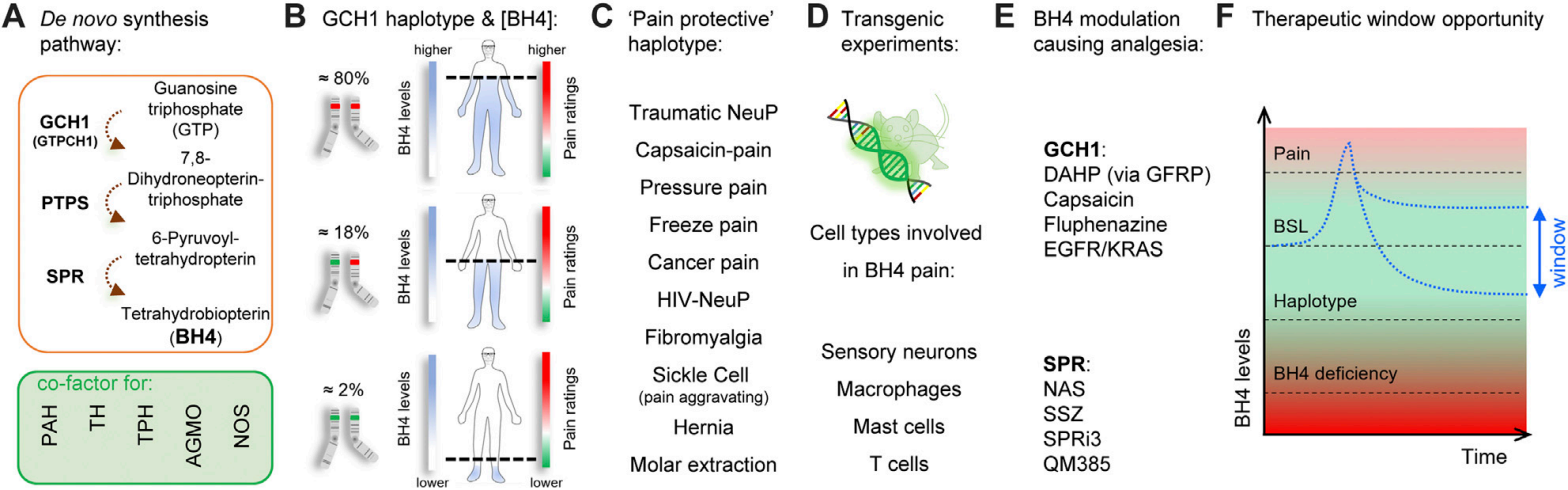
Tetrahydrobiopterin and pain. **(A)** Tetrahydrobiopterin *de novo* production pathway. BH4 is a cofactor for phenylalanine hydroxylase (PAH) (produces tyrosine), tyrosine hydroxylase (TH) (produces dopamine), tryptophan hydroxylase (TPH) (produces serotonin), alkyl glycerol monooxygenase (AGMO) (involved in lipid metabolism) and nitric oxide synthases (NOS) (produces NO). **(B)** A GCH1 haplotype is associated with reduced BH4 production and pain ratings (dashed line). **(C)** The ‘pain protective’ haplotype has been characterized in several pain conditions, most of them involving nerve injury. Note that in sickle cell disease, the pain protective haplotype is pain-aggravating. **(D)** Reporter and tissue-specific gain- or loss-of-function experiments have identified sensory neurons, macrophages, mast cells and T Cells to utilize BH4 in promoting pain. **(E)** Several agents that target GCH1 or SPR have analgesic properties in preclinical models. **(F)**, Graphical depiction showing the therapeutic window to normalize/reduce BH4 levels for pain relief. Red color indicates undesirable outcomes (pain sensitivity and side effects from BH4 deficiency) Green color and double-arrow area indicate the pain-free, side-effect-free therapeutic zone potentially offered by peripheral SPR inhibition. BSL, baseline.

Identified through unbiased gene expression profiling experiments in rats and confirmed by human genome-wide association studies (GWAS) (Tegeder et al., 2006), the role of tetrahydrobiopterin (BH4) in chronic pain has been validated by numerous clinical and preclinical studies over the last 15 years (Figures 1A-E; Latremoliere and Costigan, 2011; Latremoliere and Costigan, 2018; Costigan et al., 2012). BH4 is an essential cofactor for aromatic amino acid hydroxylases, nitric oxide synthases (NOSs), alkylglycerol monooxygenase (Werner et al., 2011). BH4 production is controlled by GTP cyclohydrolase-1 (GTPCH1; hereafter referred to as GCH1), which is the first and rate-limiting enzyme of the *de novo* BH4 biosynthesis pathway. At the clinical level, there exists a GCH1 ‘pain-protective’ haplotype, present in ∼2% of the general population, which has been confirmed in many chronic pain states, especially those that involve peripheral nerve injury (Tegeder et al., 2006). While GCH1 was identified through genetic linkage studies, the main factor influencing pain sensitivity is the ultimate amount of BH4 produced. The original GCH1 ‘pain-protective’ haplotype was associated with reduced BH4 levels and characterized in an extremely homogenous cohort of European descent (Tegeder et al., 2006). The same haplotype was later studied in an African-American population suffering from sickle-cell disease and was found to be pain-aggravating. Strikingly, in this condition the BH4 levels were higher in patients carrying the haplotype (Belfer et al., 2014), indicating BH4 levels correlate with pain sensitivity across a wide variety of painful conditions in humans.

An ideal therapeutic goal would be to block excessive BH4 production to reduce pain hypersensitivity, while preventing potential BH4 rundown avoiding associated side effects (Figure 1F). Insufficient BH4 levels can lead to reductions in synthesis of serotonin, adrenaline, noradrenaline, dopamine, nitric oxide, and impaired metabolism of glycerolethers and phenylalanine. Indeed, a typical clinical feature of BH4 deficiencies is hyperphenylalaninemia because phenylalanine hydroxylase (PAH) is lacking its cofactor to metabolize phenylalanine into tyrosine (Blau and Erlandsen, 2004; Werner et al., 2011; Himmelreich et al., 2021). While mutations affecting most enzymes of the BH4 anabolic pathways are associated with hyperphenylalaninemia (Himmelreich et al., 2021), a loss of function of sepiapterin reductase (SPR), the last enzyme in the *de novo* synthesis production pathway, does not (Bonafé et al., 2001). This is mostly due to the so-called “salvage pathways”, where several enzymes can maintain minimal levels of BH4 production when SPR is blocked, thereby limiting excessive loss of BH4 production, and preserving enough BH4 required for its cofactor activities (Bonafé et al., 2001; Iino et al., 2003; Hirakawa et al., 2009) in the liver, where phenylalanine is catabolized. Such biochemical redundancy could represent an opportunity to pharmacologically normalize BH4 levels to reduce pain hypersensitivity. Tool compound SPR inhibitors have been recently developed and reported to reduce pain hypersensitivity in mice without major side effects (Latremoliere et al., 2015; Fujita et al., 2020; Cronin et al., 2022). However, the salvage pathways vary depending on the species and tissues considered, and this needs to be taken into consideration when developing such compounds to improve the chances of translational success.

In this review, we make the case that SPR inhibition restricted to the periphery (i.e., excluded from the spinal cord and brain), is an efficacious and safe target to alleviate chronic pain. First, we describe how different cell types, that engage in BH4 overproduction and contribute to pain hypersensitivity, are themselves restricted to peripheral tissues and show their blockade is sufficient to alleviate pain. We discuss the likely safety profile of peripherally-restricted SPR inhibition based on human genetic data, the biochemical alternate routes of BH4 production in various tissues and species, and the potential pitfalls to predictive translation when using rodents. Finally, we propose and discuss possible formulation and molecular strategies to achieve peripherally-restricted, potent SPR inhibition to treat not only chronic pain but other conditions where excessive BH4 has been demonstrated to be pathological.

## 2 Targeting BH4 in the periphery is sufficient to reduce pain hypersensitivity

### 2.1 BH4 in peripheral sensory neurons

Neuropathic pain results from pathology induced by damage to peripheral sensory nerves that transmit pain (nociceptors) causing chronic pain symptoms such as hyperalgesia (increased pain sensation to a noxious stimulus), allodynia (innocuous stimuli now cause pain) and even spontaneous pain (pain in the absence of external stimuli). Conditions which can cause damage to peripheral nerves are varied and can include trauma and surgery, type 2 diabetes, infections and inflammation (HIV, shingles, leprosy), toxins (chemotherapy), autoimmune diseases, cancers, and certain hereditary conditions (Charcot-Marie-Tooth). Many animal models of peripheral neuropathy have been developed (reviewed at (Mogil, 2009; Mogil et al., 2010; Sadler et al., 2022)) to investigate changes in injured, and indeed adjacent non-injured, sensory neurons, as well as those affected by chronic inflammation, to understand their relative contribution to pain hypersensitivity.

In both rats and mice, peripheral nerve injury increases the expression of key enzymes responsible for BH4 production at the site of injury, in the dorsal root ganglia (DRG), but not in the spinal cord (Tegeder et al., 2006; Latremoliere et al., 2015). Downregulation of the gene *Gch1*, using DRG-targeted adeno-associated virus encoding small hairpin against *Gch1*, reduced nerve injury-mediated neuropathic pain hypersensitivity in rats, suggesting that peripheral inhibition of BH4 production was sufficient to alleviate pain (Kim et al., 2009). Conclusive evidence for the role of peripheral, DRG-derived BH4 in causing pain hypersensitivity after nerve injury came from genetic studies. The use of conditional *Gch1*-floxed mice crossed with two different DRG-targeted cre lines (Advillin-cre and tamoxifen-inducible Brn3A-ERt-cre) to specifically ablate *Gch1* in DRG neurons, demonstrated that blocking BH4 in DRG sensory neurons in the periphery attenuated and could reverse pain hypersensitivity in neuropathic and inflammatory pain models (Latremoliere et al., 2015). Moreover, it was shown recently that *Gch1* expression levels in cultured mouse DRG sensory neurons acted as an excellent cellular biomarker for neuropathic pain and drug screening to alleviate pain hypersensitivity (Cronin et al., 2022).

Abdominal pain is a key clinical symptom of many gastrointestinal conditions such as inflammatory bowel disease (IBD) which greatly hinders quality of life for those affected. Among the heterogenous cell types constituting the intestine are the enterochromaffin cells (ECs), a subtype of neuroendocrine cells which can detect noxious stimuli, similar to nociceptors of the DRG, and elicit pain (Mawe and Hoffman, 2013; Bellono et al., 2017; Salaga et al., 2019; Karakan et al., 2021). Among other effects, ECs can release serotonin (also known as 5-hydroxytryptamine (5-HT)) upon stimulation which then activates its cognate receptors (such as 5-HT_3 and 5-HT_4) on sensory nerve endings innervating the gut to send nociceptive signals to the spinal cord and brain. Recently, it was demonstrated that ECs are crucial drivers of visceral pain and anxiety associated with IBD which may be mediated by serotonin (Bayrer et al., 2023). As mentioned above, BH4 is an essential cofactor for tryptophan hydroxylase (TPH), an enzyme essential for the synthesis of serotonin. It is therefore tempting to speculate that peripheral SPR inhibition may alleviate the visceral pain hypersensitivity associated with IBD by reducing serotonin levels in ECs. Altogether, these studies suggest that blocking the BH4 pathway in peripheral sensory neurons, or intestinal neuroendocrine cells, would be sufficient to reduce pain hypersensitivity under a variety of chronic pain conditions.

### 2.2 BH4 in immune cells

Neuroimmunology represents an essential interplay between two powerhouse systems of the body controlling normal physiological homeostasis. However, dysregulated communication between the two systems can also wreak havoc to initiate, maintain and promote pathological conditions ranging from cancer and autoimmunity to neurodegeneration. The role of certain immune cells in regulating chronic pain—inflammatory, neuropathic and chemotherapy-induced - has been well established. Here we review the specific immune cells that engage or require BH4 for their function and are associated with pain hypersensitivity. Innate immune cells such as mast cells have been demonstrated to enhance thermal and mechanical pain hypersensitivity following nerve injury as well as under inflammatory conditions. Mast cells have been shown to have direct contact with nerve terminals via the cell adhesion molecular N-cadherin and this interaction can induce mast cell degranulation (Folgueras et al., 2009). Moreover, CGRP and substance P, two neuropeptides released by injured nerves can also induce mast cell degranulation (Green et al., 2019). Upon degranulation mast cells release several mediators involved in tissue repair but also factors such as histamine which has been shown to contribute to pain hypersensitivity in chronic cystitis and pancreatitis (Ribeiro et al., 2000; Hoogerwerf et al., 2005). Recently, we have shown that mast cell derived BH4 regulates serotonin production and release which contributes to mechanical and thermal hypersensitivity after tissue injury (https://www.biorxiv.org/content/10.1101/2023.01.24.525378v1). By genetically ablating *Gch1* specifically in mast cells, the resulting mice displayed substantially less pain-like behaviors in the hind paw incision model which serves as a proxy for post operative pain hypersensitivity. *Gch1*-deficiency in DRG neurons did not affect nociceptive responses after injury in this model thus expanding the repertoire of pain models and non-neuronal cell types, such as mast cells, in which peripheral SPR inhibition can be used to provide pain relief. Macrophages, another immune cell type of the innate immune system, infiltrate in large numbers the site of nerve injury as well as in the DRG tissue (Cui et al., 2000; Liu et al., 2000) where they produce mediators - nitric oxide (NO), TNFα, IL-8 and IL-1β - which lower pain thresholds (Thomazzi et al., 1997; Staats Pires et al., 2020).

Macrophages infiltrating injured nerves also upregulate *Gch1* expression which can lead to increased nitric oxide production (BH4 being an obligate cofactor for iNOS (also known as NOSII) activity) (Werner et al., 2011). Further investigation is necessary however to elucidate the role for macrophage-derived BH4 in neuropathic pain and whether it contributes directly to pain hypersensitivity, but it is likely that reduced BH4 overexpression in these cells could reduce pain responses.

The adaptive arm of the immune system, especially T Cells, has also been implicated in both inflammatory and neuropathic pain conditions. T Cells have been demonstrated to infiltrate injured nerves, DRG tissue as well as the spinal cord days to weeks after the nerve injury (Hu and McLachlan, 2002; Hu et al., 2007). In neuropathic nerve injury models and using T cell-deficient animals, it seems that activated T Cells promote pain hypersensitivity (Moalem et al., 2004; Cao and DeLeo, 2008; Costigan et al., 2009; Vicuña et al., 2015). In inflammatory pain models, T Cells were also shown to infiltrate after several days where they then persisted in the inflamed tissue (Ghasemlou et al., 2015). However, unlike the case of nerve injury, under inflammatory conditions T Cells may play more beneficial roles in pain relief through secretion of opioids (Boué et al., 2014; Basso et al., 2016) though other reports suggest they play no role under such conditions (Ghasemlou et al., 2015). Overall, the literature seems to point to a clear role of activated T Cells in aggravating neuropathic pain and thus represent an additional avenue for therapeutic intervention (Laumet et al., 2019). Interestingly, BH4 is synthesized in activated T Cells and SPR inhibition has been demonstrated to reduce T Cell proliferation and cytokine production which suppresses overall effector function (Cronin et al., 2018). Thus, in addition to inhibiting SPR in injured sensory neurons to combat pain hypersensitivity, an additional benefit of targeting SPR in the periphery is to also block the pathway in infiltrating, pain-promoting immune cells such as mast cells, macrophages and T Cells.

Altogether, these studies show that peripheral sensory neurons, mast cells, macrophages, intestinal enterochromaffin cells, and T Cells all engage the BH4 production pathway and contribute to abnormal pain hypersensitivity in various preclinical models of abnormal pain. Reducing BH4 overproduction in the periphery in some or all of these cell types might be sufficient to mimic the GCH1 ‘pain-protective’ haplotype and reduce pain hypersensitivity. However, because GCH1 is the rate-limiting enzyme for BH4 production pharmacological inhibition of this enzyme would be hard to titrate and could precipitate the risk of causing insufficient BH4 levels. In contrast, blocking the last enzyme of the *de novo* production pathway, sepiapterin reductase (SPR), is not necessarily associated with BH4 deficiency, thanks to the salvage pathways (Bonafé et al., 2001; Iino et al., 2003; Hirakawa et al., 2009) identified in the liver. These salvage pathways however are tissue- and species-specific and a clear understanding of inter-species differences is required to reduce pitfalls and improve the overall chances of translational success when designing strategies to target SPR for pain treatment. Below we describe the biochemical reactions that can produce BH4 from 6-pyruvoyl-tetrahydropterin which itself is a product of 6-pyruvoyl-tetrahydrobiopterin synthase (PTPS), and how they explain the salvage pathways in humans, mice and rats in the CNS and in peripheral tissues.

## 3 Targeting BH4 production via SPR in the periphery is safe

**FIGURE 2:**
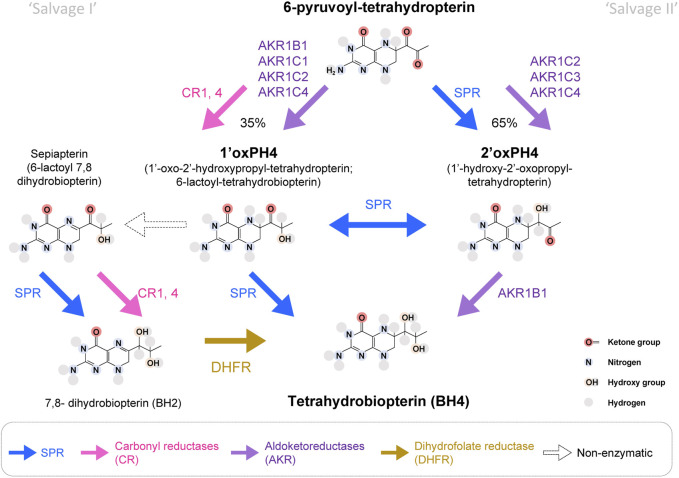
Enzymatic pathways to produce BH4 from 6-pyruvoyl-tetrahydropterin. In bold blue, SPR can produce 2′ox-PH4, isomerize it into 1′ox-PH4 and reduce it to BH4, which represents the main production route of the BH4 *de novo* synthesis pathway. SPR can also transform sepiapterin into BH2. Aldoketoreductases (AKR; purple) and carbonyl reductases (CR; pink) can also carry some of the reactions that lead to BH4. It is estimated that ∼35% of 6-pyruvoyl-tetrahydropterin will be reduced into 1′ox-PH4 by CRs, before SPR reduces it into BH4. In absence of SPR, the production of BH4 from 1′ox-PH4 represents the ‘salvage pathway I’, and the production of BH4 from 2′ox-PH4 represents the ‘salvage pathway II’. DHFR: dihydrofolate reductase (in yellow). White arrows represent pathways only possible in absence of SPR.

**FIGURE 3:**
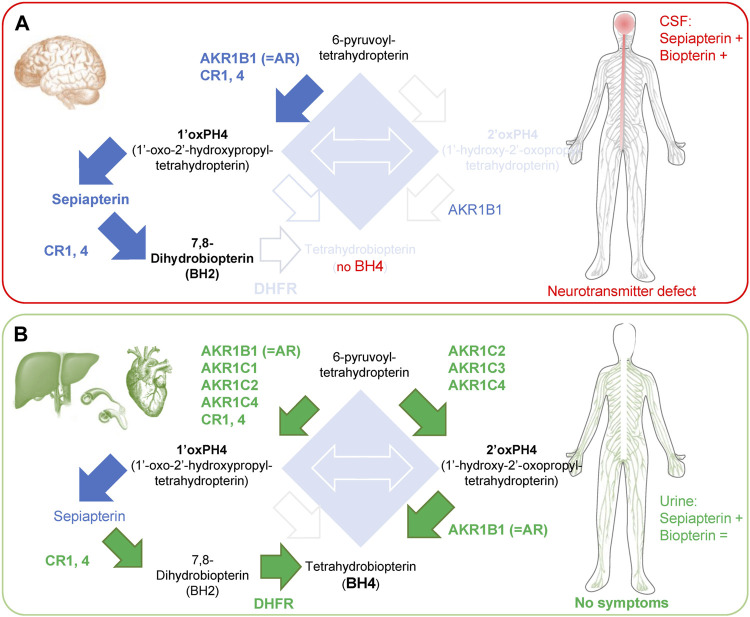
Enzymes involved in the ‘salvage pathways’ in brain and peripheral tissues of SRD. **(A)** SRD patients have limited salvage pathways in the brain, which causes side effects associated with neurotransmitter defects. In the CSF, there is an accumulation of sepiapterin and an increase in total biopterin levels, caused by the accumulation of BH2. **(B)** In contrast, organs in the periphery (liver, blood vessels and heart) express most enzymes of the ‘salvage pathways’ I and II, allowing enough BH4 production for its cofactor activities. Green arrows and text indicate BH4 production mediated by salvage pathways. Blue arrows indicate increased production of metabolite that does not lead to BH4 production. Note: high levels of 1′ox-PH4 cannot be all processed by CRs and DHFR in the liver, resulting in accumulation of sepiapterin. +, indicates an increase in levels; = , indicates unchanged levels.

While the biochemical formation of 7,8-dihydroneopterin-triphosphate by GCH1 and 6-pyruvoyl-tetrahydropterin by PTPS are totally dependent on these enzymes, the production of BH4 from 6-pyruvoyl-tetrahydropterin can be carried out by several enzymatic pathways. This partial redundancy is what allows the existence of so-called “salvage pathways”—alternate enzymatic activities that, when co-expressed within the same cell can produce BH4. Below we describe the possible enzymatic pathways from 6-pyruvoyl-tetrahydropterin to BH4 from *in vitro* studies (Figure 2). Then we discuss which combinations can explain the species- and tissue-specific profile of BH4 maintenance in SPR deficiencies (Figure 3). By describing in detail these aspects of the salvage pathways and the homeostatic maintenance of BH4, even in situations of SPR deficiency, we hope to provide a framework by which the case for pursuit of peripherally restricted SPR inhibitors can be made.

### 3.1 The many biochemical roads from 6-pyruvoyl-tetrahydropterin to BH4

6-pyruvoyl-tetrahydropterin is produced by PTPS and is the direct substrate for SPR. SPR transforms 6-pyruvoyl-tetrahydropterin into 1′-hydroxy-2′oxopropyltetrahydropterin (2′ox-PH4), followed by an isomerization into 1′oxo-2′hydroxypropyltetrahydropterin (1′ox-PH4) and its final reduction into BH4. Production of 1′ox-PH4 and 2′oxPH4 can also be carried out by several enzymes belonging to two major enzymatic superfamilies: aldoketoreductases (AKRs) and carbonyl reductases (CRs), but the transition between 1′ox-PH4 and 2′oxPH4 seems to be only carried out by SPR.

### 3.2 SPR-independent production of 1′-ox-PH4 from 6-pyruvoyl-tetrahydropterin (salvage I)

Carbonyl reductases can reduce 6-pyruvoyl-tetrahydropterin into 1′ox-PH4 with strong affinity (Milstien and Kaufman, 1989; Iino et al., 2000; Hoffmann and Maser, 2007). Several isoforms of carbonyl reductases have been discovered, which can potentially reduce 6-pyruvoyl-tetrahydropterin: two in the silk lemon, three in humans and four in mice. In humans, CBR1 and 4 have the strongest overall expression, albeit with variability between tissues (Endo et al., 2008; Hua et al., 2017; Shi and Di, 2017). In addition, the aldoketoreductases AKR1B1 (formerly known as AR), AKR1C1, C2 and C4 can also reduce 6-pyruvoyl-tetrahydropterin into 1′ox-PH4, with AKR1B1 being the most potent (Iino et al., 2003).

In the absence of SPR, 1′ox-PH4 transforms (non-enzymatically) into sepiapterin (6-lactoyl-BH2), which can then be reduced into BH2 by CRs (Iino et al., 2000; Iino et al., 2003). BH2 can be transformed into BH4 by dihydrofolate reductase (DHFR). SPR has a very strong affinity for sepiapterin (hence its name), so this metabolite typically does not accumulate when the enzyme is functional (Sueoka and Katoh, 1989). As a result, sepiapterin levels represent a reliable biomarker of SPR inhibition.

### 3.3 SPR-independent production of 2′-ox-PH4 from 6-pyruvoyl-tetrahydropterin (salvage II)

AKR1C2, C3 and C4 can produce 2′-ox-PH4. AKR1C3 (also known as 3α-hydroxysteroid dehydrogenase type 2) is more potent than AKR1C2 and 4 (Iino et al., 2003). AKR1B1 can then reduce 2′ox-PH4 (but not 1′ox-PH4) into BH4 (Hirakawa et al., 2009). AKR1B10 and AKR1B15 are two isoforms sharing sequence similarity with AKR1B1, but their ability to catalyze 2′ox-PH4 to BH4 is not known.

The most efficient salvage pathway appears to be mediated by AKR1C3 then AKR1B1, while BH4 production through sepiapterin and DHFR appears to be quite slow (Iino et al., 2003; Sawada et al., 2005). The relative tissue distribution of these enzymes will therefore determine how much BH4 can be produced when SPR is blocked.

### 3.4 Sepiapterin reductase deficiency

Sepiapterin reductase deficiency (SRD) is an inherited autosomal recessive neurotransmitter disorder first characterized 20 years ago (Bonafé et al., 2001). The onset of the disease is usually very early in childhood (as early as 3 months postnatally) and symptoms include mostly developmental delays for motor and cognitive functions, as well as oculogyric crises in the majority of cases (Zielinska et al., 2010; Leuzzi et al., 2013; AlSubhi et al., 2017; Nakagama et al., 2019). If untreated, patients will develop severe dystonia with marked diurnal fluctuations and possible hypersomnia (Neville et al., 2005; Abeling et al., 2006; Leu-Semenescu et al., 2010; Friedman et al., 2012; Koht et al., 2014; Nakagama et al., 2019). In some cases, untreated patients will also display excessive salivation and difficulties swallowing due to motor impairment (Opladen et al., 2020). Dopamine, serotonin and nitrite levels are virtually null in the cerebrospinal fluid (CSF) (Bonafé et al., 2001; Zorzi et al., 2002b; Verbeek et al., 2008; Wali et al., 2010; Leuzzi et al., 2013; AlSubhi et al., 2017; Nakagama et al., 2019), indicating a severe loss of neurotransmitter synthesis and NOS function. The lack of function of these enzymes is explained by an absence of BH4 in the CSF (Nakagama et al., 2019). Total levels of biopterin however are increased in CSF due to the accumulation of BH2 (Wali et al., 2010; Leuzzi et al., 2013; AlSubhi et al., 2017). Finally, sepiapterin levels are strongly increased in CSF confirming the absence of functional SPR (Zorzi et al., 2002a; Verbeek et al., 2008; Wali et al., 2010; Leuzzi et al., 2013; Carducci et al., 2015; AlSubhi et al., 2017). Sepiapterin can also be detected in urine of SRD patients (Zorzi et al., 2002a; Carducci et al., 2015), while biopterin levels are not changed.

Importantly, SRD is one of the rare BH4 deficiency syndromes that is not associated with hyperphenylalaninemia (Bonafé et al., 2001; Neville et al., 2005; Abeling et al., 2006; Leu-Semenescu et al., 2010)). This is because most of the enzymes required for salvage pathways (I and II) are heavily expressed in peripheral tissues such as the liver, where phenylalanine catabolism into tyrosine occurs (Bonafé et al., 2001; Iino et al., 2003; Hirakawa et al., 2009). While the majority of BH4 in the liver is maintained through the recycling pathway, consisting of quinoid dihydropteridine reductase (QDPR; also known as Dihydropteridine reductase; DHPR) and pterin-4α-carbinolamine dehydratase) (Werner et al., 2011; Himmelreich et al., 2021), a disruption of the *de novo* BH4 synthesis pathway will lead to BH4 deficiency and in most cases hyperphenylalaninemia (i.e., some GCH1 loss of function, all PTPS loss of function patients).

### 3.5 Enzymes responsible for salvage pathways in humans

The strong reduction in monoamine production without hyperphenylalaninemia in SRD patients is because human brains display low activity for both salvage I and salvage II pathways*.* AKR1C2, 3 and 4 are expressed at extremely low levels in the brain (Khanna et al., 1995; Penning et al., 2000; Hirakawa et al., 2009), so 2′ox-PH4 cannot be produced (e.g., low salvage II pathway (Hirakawa et al., 2009)). AKR1B1 and CR1, 4 are expressed in the brain, which allows the production of 1′ox-PH4 that is then oxidized non-enzymatically into sepiapterin. While sepiapterin can be in parts transformed into BH2 by CR1 and CR4, it has been proposed that the low activity of DHFR in the brain prevents the production of BH4 (Bonafé et al., 2001), which would explain the observed BH2 accumulation in CSF of SRD patients. Sepiapterin in turn also accumulates and is then secreted/released by cells, and that can be measured in the CSF (Figure 3A).

In the liver, AKR1C2, 3 and 4 isoforms and DHFR are highly expressed so BH4 can be produced from 2′ox-PH4 and BH2, respectively. This is enough to provide baseline, physiological activity of PAH in SPR-deficient patients and so, importantly, negating hyperphenylalaninemia development. A phenylalanine challenge however reveals a defect in metabolism kinetics (Abeling et al., 2006), reflecting how the salvage pathways are not as efficient as when fully functional SPR is present (Iino et al., 2000; Iino et al., 2003; Abeling et al., 2006). In the heart, DHFR levels appear low, but AKR1C2, 3 and AKR1B1 are extremely high, which could explain why no cardiovascular problems have been reported in SRD patients (Figure 3B).

### 3.6 Enzymes responsible for salvage pathways in rodents

The consequences of blocking SPR in rodents have yielded contrasting results, mostly due to the species-specific expression profile of the numerous enzymes that can contribute to the salvage pathways.

Mice lacking SPR have a defect in brain serotonin and dopamine levels but also hyperphenylalaninemia and increased blood pressure and arrhythmia (Yang et al., 2006; Takazawa et al., 2008; Sumi-Ichinose et al., 2017). The development of hyperphenylalaninemia is mostly explained by the lack of the enzyme AKR1B3 (equivalent of AKR1B1 in humans) in mouse liver (Hirakawa et al., 2009), thereby preventing the reduction of 2′ox-PH4 into BH4. As a result, only the ‘salvage I’ pathway can produce BH4, but this is insufficient for PAH baseline function (Yang et al., 2006; Takazawa et al., 2008). A recent study indicated SPR KO mice also display increased blood pressure and arrhythmia (Sumi-Ichinose et al., 2017). These changes however appear to be caused mainly by an imbalance of the sympathetic/parasympathetic tone over time, rather than any intrinsic heart dysfunction (Sumi-Ichinose et al., 2017). Transient blockade of SPR by potent inhibitors does not appear to induce changes in cardiovascular measures in mice (Latremoliere et al., 2015). Endothelial cells produce nitric oxide via endothelial nitric oxide synthase (eNOS (also known as NOSIII) that is important for vasodilatation (Bendall et al., 2014) and an abnormal balance of BH4/BH2 can lead to NOS uncoupling which results in reactive species overproduction in rodents such as peroxynitrite (ONOO^−^) and H_2_O_2_ that contribute to oxidative stress. Interestingly, in SRD patients, there are no signs of BH4/BH2 imbalance as indicated by urine biopterin measurements, which should limit the risk of NOS uncoupling in humans, even during chronic treatment.

Finally, SPR KO mice have some BH4 production in the brain (Yang et al., 2006; Takazawa et al., 2008), suggesting some alternate routes of production in this species. There are two mouse orthologues for human AKR1C3 called AKR1C6 and AKR1C18, with the latter being strongly expressed in the brain (Pratt-Hyatt et al., 2013). It is possible then that 6-pyruvoyl-tetrahydropterin is processed into BH4 to some extent in mouse brain. This level of BH4 production (∼20% WT) is not sufficient however for baseline production of monoamines in this tissue. In rat brains, both AKR1C3 and AKR1B1 are expressed (Azzarello et al., 2008), suggesting that the salvage II pathway might compensate even more for SPR inhibition in this species. Rats then could be partially ‘CNS-protected’ against BH4 deficiencies and would therefore not represent the species of choice to test for CNS side effects by SPR peripherally restricted inhibitors.

Altogether, the overall clinical profile of SRD patients, the most extreme case of SPR loss-of-function, strongly suggests that the lack of this enzyme is not associated with major peripheral symptoms owing to the presence of the salvage pathways. The main symptom caused by loss-of-function of SPR is a reduction in neurotransmitter synthesis which causes developmental delays. This can be treated well by administration of the precursors L-DOPA (l-3,4-dihydroxyphenylalanine) and 5-HTP (5-hydroxytryptophan) to elevate dopamine and serotonin levels respectively. When given during early childhood, most developmental delays can be reversed. In adult SRD patients, cessation of 5-HTP treatment causes non-motor CNS adverse effects, but no apparent peripheral side effects (Leu-Semenescu et al., 2010).

**FIGURE 4:**
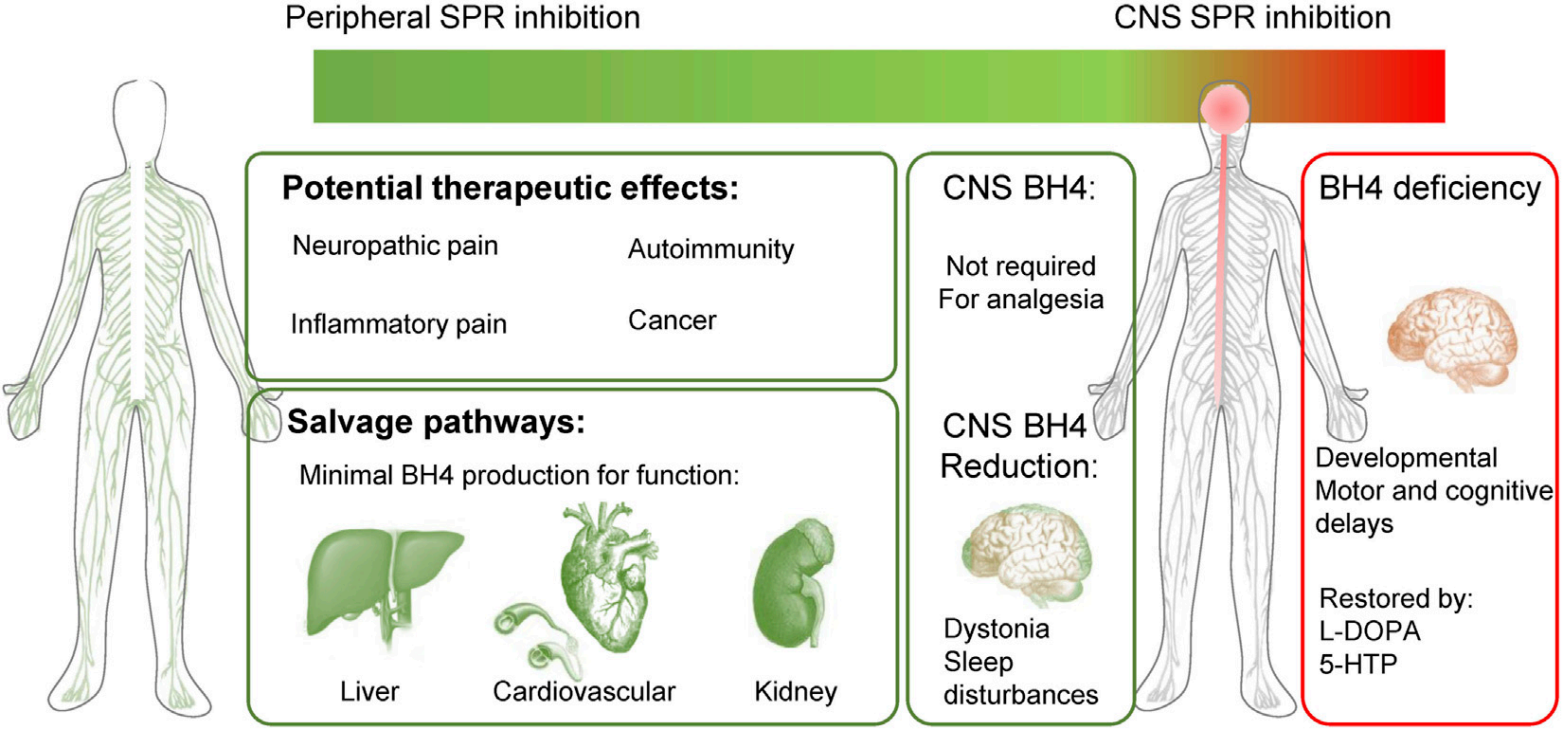
Peripherally-restricted SPR inhibition could have analgesic properties for several chronic pain conditions, without major side effects. Salvage pathways can produce minimal BH4 levels for physiological function in the liver, heart, blood vessels and kidney. SPR inhibition in the CNS is not required to modulate pain sensitivity and could cause undesirable side effects (motor problems such as dystonia, sleep disturbances). Patients with sepiapterin reductase deficiency (SRD) mostly display CNS developmental defects, which are well treated with L-DOPA and 5-HTP. Green represents a good therapeutic profile (analgesia and no side effects) and red undesirable effects.

The species differences we have highlighted are key technical points to consider when developing peripherally restricted SPR inhibitors. Indeed, the expression profile of the various enzymes within the brain, liver and cardiovascular system explains most of the discrepancies observed in SPR deficiencies between humans and rodents. The analysis of SPR deficiency analogous to a total blockade of this enzyme, reveals a very good safety profile in the periphery, and explains how some salvage pathways in the rodent brain can lead to underestimating CNS adverse effects in other species (such as dogs or humans). Because of this, measuring BH4 and monoamine levels or motor-related dysfunction is not recommended as we would predict some brain SPR inhibition will be under-estimated. However, measuring sepiapterin levels certainly represents a more sensitive biomarker for SPR inhibition (Latremoliere et al., 2015; Fujita et al., 2020) to assess brain penetrance and target engagement in preclinical species. Since SRD patients have strong circadian/sleep disturbances, early changes in sleep-wake architecture might also be interesting biomarkers of SPR inhibition in CNS. The clinical profile of SRD patients strongly suggests that peripheral inhibition, or even blockade of SPR is safe and represents an effective way to normalize BH4 levels and reduce pain hypersensitivity. Modulation of BH4 levels in the brain is not required to produce analgesia and early signs of SPR inhibition in the CNS could be mild motor and sleep disturbances (Figure 4).

## 4 Developing peripherally restricted SPR inhibitors

### 4.1 A short history of SPR inhibition

In the early 1980s and 90s, researchers, interested in feedback regulatory mechanisms of BH4 synthesis, identified N-acetyl-serotonin (NAS) as a specific binder and inhibitor of SPR (Katoh et al., 1982; Smith et al., 1992). NAS is an intermediatory natural metabolite produced from serotonin in the synthesis of melatonin and thus with increasing levels can negatively feedback on SPR to limit serotonin production. The crystallographic structure of NAS binding to SPR was resolved and showed binding of the N-acetyl and indole hydroxyl groups of NAS to SPR as important for specific inhibition (Nar et al., 1995; Auerbach et al., 1997). However, as NAS is a natural metabolite, its therapeutic use as an inhibitor of SPR is limited due to the fact that melatonin production will be affected; melatonin has diverse roles in circadian rhythm generation and sleep, as well as blood pressure regulation.

Sulfasalazine (SSZ) is an anti-inflammatory drug used to treat autoimmune conditions such as ulcerative colitis, Crohn’s disease, psoriasis, and rheumatoid arthritis. SSZ is broken down by commensal bacteria in the colon to produce two active metabolites—sulfapyridine and mesalamine—both of which have immunosuppressive effects though exact mechanisms are not fully understood. A yeast-three-hybrid system to identify drug-protein interactions uncovered a specific interaction between SSZ and SPR (Chidley et al., 2011). SSZ inhibited SPR enzymatic activity much more potently than NAS (IC_50_ 23 nm *versus* 3,100 nM). Sulfapyridine also inhibited SPR activity (IC_50_ 480 nM) while mesalamine only poorly affected SPR activity (IC_50_ 370 μM) (Chidley et al., 2011). Indeed, SSZ has been shown to have pain-relieving effects in rodent chronic pain models including neuropathic pain (Yasukochi et al., 2021) as well as diabetic neuropathy (Berti-Mattera et al., 2008). A follow-up study showed that many other anti-bacterial ‘sulfa’ drugs containing the conserved sulfonamide moiety of SSZ can inhibit SPR and this may explain their adverse CNS-related side effects (Haruki et al., 2013). However, to date, there have been no clinical trials to study the effects of SSZ on human neuropathic pain patients. This may be due to the fact that SSZ is metabolized into different bioactive components of which little is known regarding their direct targets, and that SSZ use has been linked to rare side effects including male sterility (Toovey et al., 1981; Fukushima et al., 2005) and axonal neuropathy (Essouiri et al., 2016), or due to drug repurposing/patent issues, as well as its low cell permeability and potency as a SPR/BH4 blocker (Haruki et al., 2013; Latremoliere et al., 2015). It therefore does not represent the most effective, safe way to specifically target SPR. Another established drug which only recently has been linked to SPR inhibition is Tranilast (*N*-3′,4′-dimethoxycinnamoyl-anthranilic acid), an anti-allergy compound. Tranilast is an analog of a tryptophan metabolite and demonstrated a direct reduction of SPR activity, being slightly more potent than NAS (IC_50_ 5.89 μM vs. 11.61 μM) (Moore et al., 2019). Similar to SSZ, the mechanisms of action of Tranilast are not well understood and effects have been demonstrated in blocking several processes including: mast cell degranulation, TGF signaling, matrix metalloprotease secretion, NALP3 inflammasome activation and the ion channel TRPV2. Given the potential side-effects and indeed low potency of established drugs like SSZ and Tranilast, new, specific and effective SPR inhibitors are needed.

The high-resolution crystal structure of NAS-SPR facilitated the design of N-(2-(5-hydroxy-2-methyl-1H-indol-3-yl) ethyl)-2-methoxyacetamide, which we designated SPRi3 (the third inhibitor of SPR, after NAS and SSZ) (Latremoliere et al., 2015). SPRi3 was designed based on the structure of NAS with key differences - SPRi3 has an additional methyl group at the 2-position of the indole scaffold and a methoxyacetyl group replacing the acetyl group, both modifications substantially enhancing the binding and inhibitory effect of SPRi3 on SPR (Latremoliere et al., 2015). When tested in mice, SPRi3 significantly reduced neuropathic and inflammatory pain (Latremoliere et al., 2015; Fujita et al., 2020) and confirmed sepiapterin as an excellent biomarker for SPR engagement/inhibition. A second compound was designed and developed by Quartet Medicine, Boston - a biotechnology company established by Atlas Ventures to discover and develop novel SPR inhibitors. A tool compound, QM385 is structurally distinct from SPRi3 (Cronin et al., 2018), and bound to SPR with a superior IC_50_ compared with SPRi3 and NAS, and effectively reduced BH4 levels in functional assays using stimulated mouse splenocytes as well as human peripheral blood mononuclear cells (Cronin et al., 2018). QM385 was mainly studied in the context of ameliorating various autoimmune conditions in rodent models in which BH4 plays a prominent role (Cronin et al., 2018), but has also shown substantial pain relief in a number of chronic pain models (Fujita et al., 2020). Another drug, patented by Quartet and subsequently synthesized by Amgen (United States), called Q-1195 did not show an analgesic effect in a rat neuropathic pain model (Meyer et al., 2019). The authors measured BH4 indirectly in the ipsilateral DRG of the rat model by oxidizing BH4 as well as BH2 to biopterin (due to low sample size) and showed an increase which was reduced by the SPR inhibitor. They also measured sepiapterin as a biomarker of target engagement in the ipsilateral and contralateral DRGs but these measures gave a confusing profile as Q-1195 did not significantly increase sepiapterin, suggesting that perhaps the amount of SPR engagement was not sufficient (Meyer et al., 2019).

There are also publications describing programs to design small molecule inhibitors of SPR. An Amgen (United States) study using *in silico* screening on the publicly available x-ray structure of SPR uncovered several diverse molecules which could target the substrate binding pocket of SPR (Gao et al., 2020). Another report from Grunenthal GmbH (Germany) employed fragment-based screening to identify novel binders of SPR (Alen et al., 2019). Neither study however showed cell-based nor *in vivo* work with their compounds. Though Quartet Medicine did not publish any papers in peer reviewed journals, they did file patents to cover their library of compounds. In publication number US20170096435A1, 2055 compounds were listed with *in vitro* SPR inhibitory activity and seven compounds were listed as orally active to reduce pain hypersensitivity in two rat models of traumatic nerve injury.

The evolution of SPR inhibitors has led to the discovery of several new blockers of the enzyme. Established drugs such as SSZ and Tranilast have decades of use in the clinic for specific immune-related conditions though these drugs have other suspected pharmacology and undesirable side effects have been observed in a small number of cases. The endogenous compound NAS which inhibits SPR as part of a negative feedback mechanism, displayed the least potency in terms of SPR binding and BH4 reduction in cellular systems, however served as a scaffold to design and synthesize specific SPR inhibitors such as SPRi3. Together with QM385, these new SPR blockers are extremely efficient in inhibiting SPR and show strong analgesic activity in several chronic pain models. However, their ability to cross the blood-brain-barrier (BBB) and gain access to the CNS where BH4 plays a prominent role in neurotransmitter synthesis, points to the necessity of limiting CNS SPR inhibition in the search for next-generation SPR inhibitors. Together with human genetic data indicating that near total blockade of SPR in the periphery does not cause pathological BH4 deficiency, and that the salvage pathways are relatively weak, at least in the human brain, we propose that the best profile for therapeutic SPR inhibition is a compound that blocks SPR selectively, i.e., with no affinity for other enzymes in the BH4 pathway, coupled with strict peripheral restriction.

### 4.2 SPR inhibitors with CNS exclusion and tissue specificity

**FIGURE 5:**
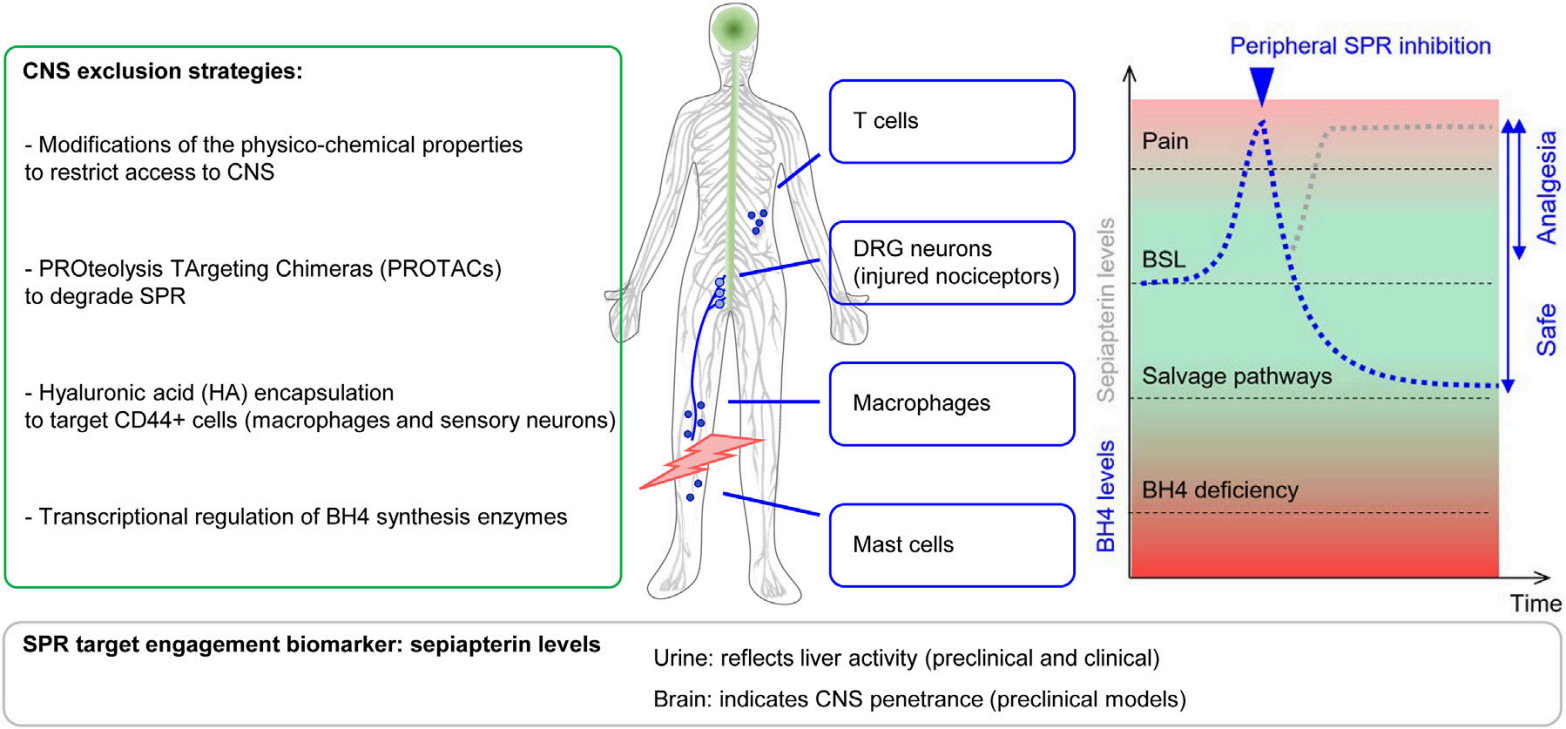
Possible strategies to produce peripherally-restricted SPR inhibitors. Left, modification of the physico-chemical properties of SPR inhibitors to promote CNS exclusion, selective tissue targeting via hyaluronic acid (HA) encapsulation, PROteolysis TArgeting Chimeras (PROTACs) and transcriptional regulators of key enzymes responsible for BH4 production. Injured peripheral sensory neurons, macrophages, mast cells and T Cells engage the BH4 production pathway in the periphery to increase pain sensitivity. Right, peripherally-restricted SPR inhibition (in blue) will reduce BH4 overproduction and pain sensitivity, while salvage pathways will allow alternate routes of BH4 production to avoid side effects associated with BH4 rundown. Bottom: urine sepiapterin levels can be used to monitor SPR target engagement to titer doses.

As is clear from the preceding sections, there is a strong rationale for our proposal that restricting the biodistribution of SPR inhibitors to the periphery will prevent the occurrence of CNS-related side-effects without loss of analgesic efficacy. Several SPR inhibitors have been shown to penetrate the CNS and it has been proposed that some of the side-effects seen with these compounds may be related to their inhibition of BH4 synthesis (Haruki et al., 2016). A blog article by Booth https://lifescivc.com/2017/11/painful-truth-successful-failure-biotech-startup/on the closure of Quartet, a biotech established by Atlas Ventures to discover and develop novel SPR inhibitors, also highlighted the problems of CNS penetration, as on-target neurologic effects in the last few days of treatment in the GLP 28-day toxicology study were sufficient to stop the entire program. It is thus abundantly clear that peripheral restriction of SPR inhibitors is essential for their safe prescription, but how can this be achieved? (Figure 5).

#### 4.2.1 CNS exclusion

In order for compounds to enter the CNS they must pass through the blood-brain-barrier (BBB) and the blood-CSF-barrier (BCSFB). The traditional approach would be to modify the physico-chemical properties of the inhibitors using standard medicinal chemistry. A lot of work has focused on identifying the most important properties of a molecule that regulate CNS penetration. The ‘Lipinsky Rule of 5’ outlines five key properties of molecules that define the extent to which a compound will penetrate the CNS and these properties are molecular weight, lipophilicity, polar surface area, hydrogen bonding, and charge (Lipinski et al., 2012). Medicinal chemists wanting to make compounds that penetrate the CNS use the rule of five to try to create molecules that have a balance of properties that favor CNS penetration, while maintaining target binding and other relevant properties for a clinical drug candidate. For example, it is a general rule that if there are fewer than 5 hydrogen bond donors and 10 hydrogen bond acceptors in a molecule or when the molecular weight is lower than 500 or the calculated log P (C log P) is between 1 and 3 then CNS penetration is more favorable. However, these are not the only issues determining CNS penetration or lack of it. For instance the compound should not be a substrate for efflux transporters such as p-glycoproteins, they should not be extremely highly bound to plasma proteins (or if they are then the compound needs to have a very high affinity for the target as only a small fraction of unbound compound will be available for pharmacological activity) and they should typically not be subject to a high rate of systemic clearance.

While modification of the physico-chemical properties of small molecule inhibitors can restrict the passage of them across the BBB and BCSFB, such traditional approaches typically only result in restriction of CNS penetration to maybe 5% of the plasma concentration, while still maintaining desirable “drug like” properties. This means that if the target receptor resides in the CNS, as well as the periphery, and the compound has high affinity for the receptor, then CNS penetration will become the dose limiting factor if the pharmacological effect in the CNS is an undesirable side-effect. As well as the difficulty in reducing CNS penetration to levels low enough to have no efficacy in the brain, traditional medicinal chemistry approaches are not typically able to deliver tissue specificity and other approaches are required.

#### 4.2.2 Selective tissue targeting of SPR inhibitors

An ideal therapeutic treatment for pain would be an agent that targeted the injured or inflamed tissues while minimizing interaction with unaffected tissues thereby optimizing efficacy and reducing side-effects. Knowledge of the regional distribution of receptors, channels and other target proteins is a key driver to understanding where a drug needs to be delivered for efficacy. For example, knowing that a target protein is selectively located on sensory neurons innervating a knee joint would increase the rationale for developing a molecule for osteoarthritis. But how to get the drug to the site of action without affecting other tissues where the target is located? Direct delivery approaches exist in the pain field, such as direct injection of local anesthetics to induce a nerve block, but such techniques are only suitable in specific situations and are not associated with treatment of pain outside the physician’s room. SPR itself is widely expressed in the periphery and the CNS and as such, systemic administration does not result in inhibition of SPR in only one compartment, thus there is a potential for side-effects related to reductions in dopamine in the CNS, as mentioned in earlier sections. However, since SPR inhibitors are likely to have efficacy in the treatment of chronic pain, systemic administration is a highly desirable method by which to deliver such compounds as patient compliance is far greater than having to endure injections into joints, for example,. One exciting prospect for delivering SPR inhibitors to specific tissues of interest, while also excluding them from the CNS is in combination with hyaluronic acid (HA), and possibly more specifically as nanoparticles encapsulating a small molecule SPR inhibitor. As explained below, the use of HA to target small molecule SPR inhibitors to injured tissues or inflammatory cells by exploiting the increased expression of CD44 in such circumstances, offers the possibility to avoid inhibiting BH4 production in tissues possessing the salvage pathway such as liver, kidney, and cardiovascular system.

#### 4.2.3 HA encapsulation to achieve selective tissue delivery

Hyaluronic acid (HA) is a naturally occurring polymer and more specifically is a linear, unbranched polysaccharide made up of repeating units of disaccharides of D-glucuronic acid and N-acetyl-D-glucosamine. It is synthesized by three transmembrane enzymes (hyaluronan synthases 1, 2 and 3) and it is degraded by two intracellular endoglycosidases (hyaluronidases 1 and 2)—reviewed at (Fallacara et al., 2018). The high degree of biocompatibility and biodegradability makes HA a potentially useful drug delivery molecule (Harrer et al., 2021). HA binds to the cellular adhesion molecule CD44 which is located on the surface of cell membranes and of relevance to the treatment of pain CD44 is upregulated on nociceptors following nerve injury (Renthal et al., 2020) and on immune cells during inflammation (Johnson and Ruffell, 2009) providing the potential for preferential targeting of injured and inflamed tissues. Once bound to CD44, HA undergoes endocytosis and initial degradation by hyaluronidase 2 and then once in the cytoplasm it is further degraded by hyaluronidase 1 upon which the cargo is released.

**Table:** 

Hyaluronic acid as a drug delivery vehicle
Advantages
Biocompatible, non-toxic and non-inflammatory
Biodegradable
Selectively targets tissues where CD44 is over-expressed
Can provide protection to its ‘cargo’
Enables delivery of poorly soluble, hydrophobic drugs
Effectively restricts drug delivery to the periphery
Chemical modification can improve half-life and selectivity of targeting to specific tissues, e.g., Endogenous inspired biomineral-installed hyaluronan nanoparticles as pH-responsive carrier of methotrexate for rheumatoid arthritis

Disadvantages
Rapidly cleared from the blood (T1/2 is about 6 min) circulation

HA is a major component of the extracellular matrix and as such has been used alone to treat osteoarthritis for more than 20 years. There are a number of products on the market that are approved for injection into the knee joint to treat pain in osteoarthritis but the mechanism by which it reduces pain is unknown. For some, the HA is avian-derived such as Hyalgan (sodium hyaluronan from rooster combs) and Orthovisc (sodium hyaluronan from rooster combs) and some bacterial-derived such as Trivisc, Euflexxa, Monovisc. HA can be given orally but to date there are no approved oral formulations of HA and they are sold as supplements. Given the lack of understanding on how HA can reduce pain in joints, there have been several recent studies investigating the effect of HA itself on pain in rats. For example, high molecular weight HA was able to reduce mechanical hypersensitivity in a model of chemotherapy-induced neuropathic pain and also a model of inflammatory pain induced by carrageenan (Ferrari et al., 2016). Further work from the same laboratory (Ferrari et al., 2018; Bonet et al., 2020) showed in rats that intra-thecal injection of antisense for CD44 and also injection of the CD44 inhibitor A5G27 blocked the effects of high molecular weight HA, thereby indicating that one mechanism is through interaction with CD44. Interestingly the authors also showed that low molecular weight HA was pro-inflammatory. However, in the clinic, there is debate as to whether the efficacy is actually different between high and low molecular weight HA. It was reported that the results of a comparative trial in osteoarthritic patients of GO-ON (800–1,500 KDa HA) and Hyalgan (500-730 KDa) that patients treated with GO-ON for 6 months had a superior response to those on Hyalgan (Berenbaum et al., 2012). However, others have found both high and low molecular weight hyaluronic acid significantly improved pain measures for late-stage knee OA patients (Karatosun et al., 2005).

While the effects of HA alone are of interest, with respect to SPR inhibitors, the properties of HA as a drug delivery molecule are of far more relevance because it may help to deliver compounds to tissues in the periphery that express CD44 and exclude delivery to the CNS through size exclusion. Small molecules can either be conjugated to HA or encapsulated within HA micelles or nanoparticles (see (Lee et al., 2020) for excellent review). There are examples of analgesics conjugated to HA such as HA-bupivacaine (Gianolio et al., 2005), HA-methotrexate (Tamura et al., 2016), HA-diclofenac (Nishida et al., 2021) however all such complexes are administered either by local intra-articular injection or topically since the molecules do not survive systemic administration. Therefore, conjugation to HA is of less interest with respect to SPR inhibition unless the HA-SPR inhibitor complex can be made sufficiently stable to resist metabolism prior to endocytosis in the cell of interest because release of the small molecule inhibitor into the systemic circulation would result in the side-effects discussed earlier. Encapsulation on the other hand, would enable the payload of the SPR inhibitor to be delivered to the intracellular compartment without the confound of systemic distribution. Systemic administration of such nanoparticles would not lead to CNS penetration since HA is such a large molecule. Using radiolabeled high molecular weight HA it was reported that passage across the BBB and BCSFB was negligible as it could not be detected in brains of dogs or rats after oral ingestion of a bolus dose of 99mtechnetium-labeled, high-molecular-weight hyaluronan (Balogh et al., 2008). There are now many published examples of HA nanoparticles, particularly in the field of cancer but also relevant to this article on inhibiting the BH4 pathway and treatment of chronic pain. In a very elegant study it was shown that methotrexate (a DHFR inhibitor used for the treatment of rheumatoid arthritis) could be packaged into biomineral-installed hyaluronan nanoparticles (Alam et al., 2017). The nanoparticles were internalized into macrophages via receptor-mediated endocytosis and their cargo of methotrexate released into the cytosol. Further experiments showed that the nanoparticles accumulated in the paws and joints of rats with collagen-induced arthritis after intraperitoneal administration and that a high dose of methotrexate (50 mg/kg), toxic to mice after systemic administration, did not have a toxic effect, demonstrating an increased level of safety through the specific delivery of methotrexate to the joints. Such work demonstrates the potential of HA-nanocarriers as a mode for delivering SPR inhibitors safely to peripheral neurons and immune cells expressing CD44.

#### 4.2.4 PROTACs as peripheral restricted SPR therapeutic

In the last 2 decades,** PRO**teolysis **TA**rgeting **C**himeras (PROTACs) have emerged as a promising technology for specific protein degradation and are now, 20 years after their initial discovery, entering Phase II clinical trials for breast (NCT03888612) and prostate (NCT04072952) cancer (Sakamoto et al., 2001; Békés et al., 2022). PROTACs are bifunctional molecules which at one end connect to a protein of interest and, at the other end, bind and recruit an E3 ubiquitin ligase (Hanzl and Winter 2020). A variable linger region separates these two functional ends. A PROTAC promotes a ternary structure between itself, a protein of interest and the ubiquitin ligase which, due to the close proximity, results in ubiquitination of the protein of interest which targets it for subsequent degradation via the ubiquitin-proteosome system. The power and versatility of PROTAC targeting lies in the fact that specific binders are enough to target a given protein of interest and not an exclusive ligand to an enzymatic or catalytic active site.

PROTACs show poor blood-brain-barrier (BBB) permeability due to their high molecular weight (generally >1,000 Da) (Sun et al., 2019; Scott et al., 2020). Moreover, the BBB and cell permeability can vary depending on the linker region between the two functional ends of the PROTAC. Linker optimization determines the efficiency of ubiquitination as the distance between the protein of interest and E3 ligase affects the flexibility and proximity of ternary complex formation and function (Hanzl and Winter 2020). The linker length is also an important prerequisite for cell and BBB permeability. The cerebron (CRBN) and von Hippel-Lindau (VHL) ligands are the two most used E3 ligases when designing PROTACs due to their ubiquitous and high expression levels in various cell types. However, the possibility exists to design more cell type specific E3 ligase binders to limit PROTAC function to desired cell types or tissues of interest such as the DRG sensory neuronal subtypes involved in neuropathic pain. Indeed, a more comprehensive profile of E3 ligase expression in various tissues under normal and pathological conditions may be needed to design efficient PROTACs and target them to the DRG or injured neurons.

Unlike, small molecular inhibition of SPR through its enzymatic domain, PROTAC degradation results in degradation of the entire protein, including its enzymatic functions but also any non-enzymatic functions. Furthermore, PROTAC molecules dissociate after polyubiquitination and are thus free to repeat the targeting steps; thus, operating at relatively low doses so to limit drug resistance and toxicity. Moreover, as PROTAC results in protein degradation, the specific ‘inhibition’ lasts longer than traditional small molecule inhibition and the half-life depends on the half-life of the target protein in any given tissue as defined by its transcription and/or translation kinetics. However of course, these benefits can be seen as a double-edged sword depending on the protein of interest; prolonged degradation maybe have undesirable effects whereby more transient inhibition is better; similarly, specifically targeting a catalytic site of an enzyme, for example, while sparing non-enzymatic functions can offer optimal and safer effects for certain proteins of interest. PROTACs consisting of a ternary complex between the protein of interest and the E3 ligase can be susceptible to a phenomenon called the “Hook effect” which occurs at high PROTAC concentrations whereby binary complex (protein of interest-PROTAC and PROTAC-E3 ligase) formation is predominant and thus prevents functional ternary complex formation resulting in loss of degradation effects. With SPR however, human genetics again guides us that a loss of SPR protein in the periphery should be very well tolerated in humans and that the ‘Hook effect’ would actually still lead to an inhibition of SPR activity (without degradation) and so again provide peripheralized SPR blockage.

#### 4.2.5 Targeting BH4 for pain relief through transcriptional regulation of key enzymes

Since the early 90s, pain therapeutics, and indeed the majority of drug discovery, have focused on target-based screening; using target-dependent assays, structural modeling and computational analyses to discover and modify molecules which specifically interact with target proteins of interest. A similar approach was utilized to design and synthesize SPRi3 and QM385, both of which target the catalytic domain of SPR. However, before this target-based approach, phenotypic screening was prevalent often with very limited information on the underlying mechanisms involved in the disease in question, and in which no ‘targets’ were known. Indeed, the success of these phenotypic screens over the more precision targeted screen is revealed by the fact that the majority of first-in-class drugs actually came from phenotypic screens (Swinney and Anthony, 2011; Swinney, 2013).

It is estimated that a new drug, from inception to launch, can cost up to $1bn, and takes approximately 15 years (Lage et al., 2018). It is a complex, difficult and costly venture in which the majority of original approaches and findings fall by the wayside. Drug repurposing is essentially using a drug for a purpose outside the scope of the original medical indication. The advantages of using repurposed drugs lies with their ‘de-risked’ profile, shorter development time and greatly reduced costs—these drugs have already been through vigorous pre-clinical and human safety assessments and formulation development. An example of a repurposed drug includes sildenafil, which was originally developed as an anti-hypertensive drug, but through retrospective clinical analysis was repurposed as a treatment for erectile dysfunction, with annual sales of up to 2 billion dollars (Lipman, 2015). Zidovudine, also known as azidothymidine (AZT), was used to treat cancers until *in vitro* screening of drug libraries found it to be highly effective against HIV and it was the first FDA-approved anti-HIV drug (Langtry and Campoli-Richards, 1989). Aspirin is another example which was historically used as only as an analgesic but was recently found to be effective in treating cardiovascular disease and colorectal cancers (Hybiak et al., 2020). An additional benefit of certain established drugs is that many have been biologically annotated, with their targets and underlying mechanism of actions elucidated.

Recently, we setup a BH4-based phenotypic screening approach to identify annotated and FDA-approved compounds which may block BH4 upregulation in injured, primary mouse DRG sensory neurons and so repurpose candidates as novel analgesics (Cronin et al., 2022). As we have mentioned above, BH4 levels are increased in injured nerves which contribute to pain hypersensitivity (Tegeder et al., 2006; Latremoliere et al., 2015). This increase in BH4 synthesis is primarily driven by the *de novo* rate-limiting enzyme, *Gch1*. Therefore, culturing and screening compounds against GFP expression levels in neurons from *Gch1*-GFP reporter transgenic mouse line allowed us to identify those which reduced the *Gch1* upregulation upon nerve injury (using GFP expression as a proxy for endogenous *Gch1* expression). Some of the compounds identified have previously been characterized as compounds which reduce pain hypersensitivity such as capsaicin (Anand and Bley, 2011; Peppin and Pappagallo, 2014) and our data suggests that capsaicin-dependent analgesia may be mediated through modulation of *Gch1* expression in peripheral sensory neurons. Interestingly, we uncovered an unexpected role for the anti-psychotic, fluphenazine hydrochloride in reducing *Gch1* expression and BH4 levels after nerve injury not only in cultured neurons but also *in vivo* in injured nerves from neuropathic pain-induced mice (Cronin et al., 2022). Importantly, fluphenazine also showed effective pain-relieving effects in mice with neuropathic pain at a dose similar to those taken by patients (Cronin et al., 2022). Indeed, there are additional findings for an analgesic effect of fluphenazine treatment in reducing mechanical allodynia in rodent neuropathic pain models (Khalilzadeh et al., 2018). Fluphenazine is an anti-psychotic used to treat schizophrenia by blocking post-synaptic D2 dopamine receptors as well as alpha-1 adrenergic receptors. The molecule has a long half-life (15–30 h) and an oral bioavailability of 40%–50%. However, common side-effects of fluphenazine treatment include movement disorders (akathisia and dyskinesia), anxiety, and depression (Knights et al., 1979; Csernansky et al., 1981). As BH4 is also needed by tyrosine hydroxylase and tryptophan hydroxylase for production of dopamine and serotonin, respectively, these effects may point to GCH1 reduction in certain brain regions upon fluphenazine treatment. Thus, a peripherally restricted form of fluphenazine that abrogates elevated GCH1 and BH4 levels in peripheral neurons after injury, could be used to lower pain sensitivity without the accompaniment of CNS side effects.

Our successful approach to targeting the transcriptional program of *Gch1* to block *de novo* BH4 synthesis could also be applied to *Spr*. We have previously shown that *Spr* expression is also increased in injured nerves after neuropathic pain in mice (Latremoliere et al., 2015) and thus may also represent an excellent target to screen against this upregulation of transcription upon nerve injury. Moreover, it should be noted that using annotated chemical libraries also provides the additional advantage of uncovering novel biology with respect to those pathways and signals that control *Spr* expression.

## 5 Peripheral SPR inhibition for autoimmunity and cancer

As mentioned above, whether it be with small molecule inhibitors, protein degraders, or targeting its transcriptional regulation, it is necessary to keep SPR inhibition in the periphery for effective and safe pain relief to abrogate any CNS-related side effects associated with BH4-dependent synthesis of essential neurotransmitters. However, in addition to reducing pain, blocking BH4 through peripheral SPR inhibition would be beneficial for several other pathological conditions. We and others have demonstrated that activated T Cells synthesize BH4 which then aids in the increased metabolic needs of the cell to elicit effector functions (Chen et al., 2011; Cronin et al., 2018). By targeting BH4 production through SPR inhibition, the T Cells could not become fully activated. Moreover, SPR inhibition *in vivo* resulted in reduced severity of T cell-mediated inflammation in models of colitis, allergic hypersensitivity and multiple sclerosis (Cronin et al., 2018; Schmitz et al., 2021), thus highlighting the potential of peripheral SPR inhibition in conditions where over-zealous T Cells are fighting against one’s own body. Indeed, SSZ is an anti-inflammatory drug used therapeutically in several autoimmune conditions such as ulcerative colitis, psoriasis, and rheumatoid arthritis. SSZ has been shown to block IL-12 in macrophages and diminish T Cell responses (Kang et al., 1999; Wang et al., 2010). Although the underlying anti-inflammatory mechanisms of SSZ have not been fully elucidated, there are numerous reports demonstrating various effects on mitochondrial function, NF-kB survival signaling as well as on the cystine/glutamate transporter (Wahl et al., 1998; Liptay et al., 1999; Gout et al., 2001). However, a recent study showing a direct effect of SSZ (and its metabolite sulfapyridine) on SPR inhibition (Chidley et al., 2011; Yang et al., 2015) may also explain these anti-inflammatory effects in a BH4-dependent context, highlighting the therapeutic value of specifically targeting SPR to treat autoimmune conditions from the immune perspective. Interestingly, many autoimmune conditions such as IBD, arthritis and psoriasis also elicit severe pain and so targeting SPR in the periphery has the dual effect of reducing inflammation as well as the pain directly.

Recently, there have been several reports showing how SPR promotes cancer progression in neuroblastoma (Lange et al., 2014; Yco et al., 2015), hepatocelluar carcinoma (Wu et al., 2020), as well as breast (Zhang et al., 2020) and ovarian (Cho et al., 2011) cancers. Indeed, *SPR* expression is significantly correlated to unfavorable neuroblastoma characteristics such as oncogenic MYC amplification and increased aggressiveness (Yco et al., 2015). We have also reported that EGFR/KRAS activation drives BH4 production in lung cancer and that blocking EGFR signaling reduces BH4 (Cronin et al., 2022). Mutations in the *RAS* genes are among the most mutated genes associated with cancer (mutated in 90% of pancreatic, 35% of lung and 45% of colon cancers) and in particular *KRAS*, is the isoform prevalently mutated in lung cancers (Prior et al., 2012). Blocking EGFR/KRAS signals is a major goal for cancer therapy and many new therapeutic approaches are currently being designed to target EGFR/KRAS activation. Intriguingly, when we genetically ablated the BH4 *de novo* pathway in *Kras*-driven cancer cells *in vivo*, the resulting tumors were smaller, and the mice survived substantially longer (Cronin et al., 2022). Some of these studies link the effects of SPR enzymatic inhibition to increased ROS levels exerting anti-proliferative and pro-apoptotic effects on the cancer cell lines, while others point to non-enzymatic functions of SPR in promoting cancer cell apoptosis (Wu et al., 2020). BH4 has also been directly associated as being protective against a relatively new type of cell death called ferroptosis (Dixon et al., 2012; Kraft et al., 2020). Ferroptosis is an iron-dependent form of cell death which is associated with high levels of ROS but lacks typical features of apoptosis such as cytochrome c release and chromatin fragmentation. It can also be blocked by iron chelation or by blocking iron cellular uptake (Dixon et al., 2012). Ferroptosis suppression is widely employed by a variety of cancers to escape cell death as they consume higher amounts of iron than healthy cells (Zhang et al., 2022). Thus, there is mounting evidence to suggest that ferroptosis induction plays a significant role in tumor rejection in radiotherapy and chemotherapy (Ma et al., 2016). Moreover, recent research has demonstrated that triple-negative breast cancer, which is the most aggressive type of breast cancer, is susceptible to ferroptosis (Verma et al., 2020) and that ferroptosis induction in cancer renders the cancer cells more susceptible to immunotherapy treatment (Wang et al., 2019; Zhao et al., 2022). Intriguingly, a genome wide CRISPR activation screen identified GCH1/BH4 as protective against ferroptosis (Kraft et al., 2020) and that blocking BH4 synthesis in cancer cells can sensitize them to cell death (Soula et al., 2020; Hu et al., 2022). Interestingly, compounds which we now know target BH4 synthesis have also shown anti-cancer properties (Haruki et al., 2013; Cronin et al., 2022). Fluphenazine exerts strong inhibitory effects on numerous cancer models including lung, breast, colon, liver and brain cancers (Hait et al., 1994; Klutzny et al., 2017; Xu et al., 2019). Sulfasalazine (SSZ) inhibits human neuroblastoma cell growth (Yco et al., 2015) as well as showing anti-proliferative effects in gastric, pancreatic and lung cancer models (Lay et al., 2007; Lo et al., 2010; Zhuang et al., 2021). These above observations open potential new avenues for cancer and autoimmune therapeutic research based on peripheral SPR inhibition.

## 6 Conclusion

We have described what we see as the therapeutic potential for peripherally restricted SPR inhibitors as non-opioid chronic pain therapeutics as well as for treatments of certain autoimmune diseases and forms of cancer. We have described how the salvage pathways differ across tissue types, and indeed species, highlighting the potential for issues with translation to the clinic. We have also suggested possible molecular approaches to restrict SPR inhibition to the periphery by encapsulation in HA nanoparticles or as part of a PROTAC strategy. Finally, we have described how a novel screening approach might be envisioned to discover novel SPR inhibitors by developing a *Spr*-GFP reporter transgenic mouse line and using GFP expression *in vitro* as a proxy for endogenous *Spr* expression. Given these exciting possibilities, we hope that future developments in the field of SPR inhibition will yield novel, safe therapeutic treatments for chronic pain and other clinical ailments.
